# Physicochemical and Functional Properties of *Citrullus mucosospermus*, *Citroides*, and *Moringa oleifera* Seeds’ Hydrocolloids

**DOI:** 10.3390/foods13071131

**Published:** 2024-04-08

**Authors:** Olakunbi Olubi, Anthony Obilana, Nsenda Tshilumbu, Veruscha Fester, Victoria Jideani

**Affiliations:** 1 Department of Food Science and Technology, Faculty of Applied Sciences, Cape Peninsula University of Technology, Cape Town 7535, South Africa; olubio@cput.ac.za (O.O.); obilanaa@cput.ac.za (A.O.); 2 Flow Process & Rheology Centre, Faculty of Engineering & the Built Environment, Cape Peninsula University of Technology, Cape Town 8000, South Africa; tshilumbun@cput.ac.za (N.T.); festerv@cput.ac.za (V.F.)

**Keywords:** hydrocolloid, *Citrullus mucosospermus*, *Citroides*, *Moringa oleifera*, physicochemical properties, functional properties, *lanatus citroides*

## Abstract

Hydrocolloids form gel-like structures when dispersed in water and have garnered significant attention for their diverse applications in food, pharmaceuticals, and other industries. The extraction of hydrocolloids from natural sources, such as seeds, presents an intriguing avenue due to the potential diversity in composition and functionality. Utilising seeds from *Citrullus lanatus mucosospermus*, *lanatus citroides*, and *Moringa* aligns with the growing demand for natural and sustainable ingredients in various industries. This research investigated hydrocolloids extracted from *Citrullus mucosospermus* (CMS), *lanatus citroides*, and *Moringa oleifera* seeds, highlighting their versatile physicochemical and functional attributes. Hydrocolloids were extracted from the seeds and subjected to analysis of their proximate composition, particle size distribution, and interfacial tension using the hot water extraction method. Protein content variation was observed among the raw oilseed (CMS, *Citroides*, and *Moringa oleifera*) flours. The protein content of the hydrocolloids surpassed that of raw oilseeds, significantly enhancing the amino acid profile. Furthermore, the hydrocolloid ash contents ranged from 4.09% to 6.52% *w*/*w* dry weight, coupled with low fat levels. The particle size distribution revealed predominantly fine particles with a narrow size distribution. All three hydrocolloids demonstrated remarkable oil- and water-holding capacities, highlighting their suitability for efficient stabilisation and emulsification in food formulations. These findings suggest the potential utilisation of these hydrocolloids as valuable ingredients across a spectrum of applications, encompassing food, pharmaceuticals, and industry, thus contributing to the development of sustainable and functional products. The unique attributes presented herein mark a noteworthy advancement in the understanding and application of novel hydrocolloids from CMS, *Citroides*, and *Moringa oleifera*.

## 1. Introduction

Hydrocolloids from plant sources offer immense nutritional and functional properties, as found in some cucurbit species, namely the *Citrullus mucosospermus* and *caffer* varieties of melon plants. Within the Benincaseae family, Citrullus is a member of the subfamily Cucurbitoideae. Four species comprise this group, two native to Namibia (*rehmii* and *ecrihosus*) [1]. However, *mucosospermus* and *colonynthis* are more common in West Africa and locally called egusi seed. The Kalahari region species is known locally as makataan and is found in the southern parts of Africa [2]. *Citrullus mucosospermus* seed, locally known as egusi seed, a wild member of the gourd family [2], has limitless potential for worldwide use in the food industry [3,4]. Egusi grows and reaches maturation between 120 and 150 days at an ideal temperature of 26–37 °C in any country with autumn/fall and summer [5].

The watermelon species *C. lanatus*, formerly *Citrullus vulgarisor* or *colocynthis*, is divided into subspecies. Subsp. Vulgaris is the most widely grown cultivar for edible pulp and subsp. *mucosospermus* grows in West Africa and is harvested as a wild, semi-cultivated, or cultivated melon. The egusi melon fatty acids are high in conjugated linoleic acid (CLA). CLA is an omega-6 fatty acid often used as a supplement for weight loss, bodybuilding, and diabetes [6]. Last is *C lanatus caffer*, comprising two varieties differentiated by the bitterness of their pulp. *Caffer* is predominant in South Africa, with the bitter pulp variety locally known as tsamma, karkoer, and bitterboela [1]. The variety with a tasteless pulp is var. *citroides*, locally called citron melon or makataan [2]. Makataan melons are under cultivation in South Africa [7]. Makataan seed can be dehulled, roasted, or eaten as it is, or its seeds can be cold-pressed to obtain a nutritious oil (68% *w*/*w* dry weight) high in omega-6 fatty acids [2].

The potential of makataan melon seeds is yet to be explored. This seed’s sole industrial application, called ‘Kalahari seed’, involves cold-pressing to extract oil [7,8]. Limited information exists regarding its nutritional and functional attributes and the potential hydrocolloid properties of its flour. Similar unexplored properties have been observed in *Moringa oleifera* seeds (MOLSs) [8], specifically as a thickening agent. The coagulation properties of MOLSs warrant increased scientific attention, with studies indicating the involvement of a soluble protein serving as a natural cationic polyelectrolyte responsible for their coagulant characteristics. The coagulant potential may enhance the technological features of food products requiring improved rheological properties [9].

Addressing food safety concerns is paramount when incorporating natural ingredients into food formulations. Despite limited research on the use of MOLSs in food applications, existing evidence suggests their safety and potential health benefits [10]. Given their substantial protein content (42–45% *w*/*w* dry weight), comparable to MOLSs and egusi seeds, exploring the diverse applications for makataan melon seed has become imperative. This revelation hints at the emergence of a novel and nutritious hydrocolloid with promising implications for consumers.

Because egusi seeds have 30% *w*/*w* dry-weight pure protein and 50% *w*/*w* dry-weight edible oil as their nutrients, they are a valuable dietary supplement [11] and a potential hydrocolloid in the food industry. Hydrocolloids are materials that form colloids with water and comprise various proteins and polysaccharides frequently utilised in the food sector [12]. Uruapka [13] found that 6% (*w*/*v*) egusi flour was sufficient to produce properly cross-linked networks (G0 8724 Pa), confirming the use of egusi as a hydrocolloid, which is still not widely used. The rheological data of egusi gels, prepared at varying protein concentrations (3, 6, 10, and 20%, *w*/*v*) in 0.15 M NaCl, has been studied.

The critical observation is that there was evidence of properly cross-linked networks when 6% (*w*/*v*) of egusi flour was used in the gel. Cross-linking refers to bonds between polymer chains, contributing to the gel’s structure and strength. The specific rheological property mentioned by Uruapka [13] is the storage modulus (G’, of 8724 Pascal (Pa). The storage modulus determines how well a material retains elastic energy during deformation. This finding is significant because it confirms that a 6% concentration of egusi flour is sufficient to produce well-structured gels with cross-linked solid networks. This suggests that egusi has hydrocolloid properties and potential applications as a thickening or gelling agent. However, egusi has yet to be widely adopted or utilised in industrial processes, possibly due to limited commercialisation or technological development.

A notable advancement in the food hydrocolloid industry regards thickening, gelling, emulsifying, and water binding [8]. The hydrocolloid properties of egusi seeds have yet to be explored despite their use in regional soups as a thickener and emulsifier [11]. Our objective was to extract hydrocolloids from the three oil seeds using the hot water extraction method while considering the preservation of the hydrocolloid properties and profiling their physicochemical and functional properties.

## 2. Materials and Methods

### 2.1. Sources of Materials and Equipment

Dehulled egusi was purchased from a local seed store in Cape Town, and MOLS was purchased from SupaNutri Pty Ltd., Graaf-Reinet, in South Africa. Makataan fruits were purchased from the Cape Town fruit and vegetable market. All chemical reagents were purchased from Merck, South Africa. All equipment was from the Flow Process and Rheology Centre at the Cape Peninsula University of Technology, Cape Town, South Africa.

### 2.2. Makataan, Moringa, and Egusi Seed Preparation and Defatting

The seeds of makataan were obtained by cutting the Makataan fruit and removing the embedded seeds. For every 800 g of seeds, 500 mL of boiled water containing 30 g of sodium chloride was added, and the mixture was allowed to soak for 6 h. After washing, the brine was decanted, and the seeds were air-dried for 2 h. The seeds were dehulled and milled using a roller mill dehuller (Afrimill-M500H Grain Hand Mill, Foreshore, Cape Town, South Africa). Following dehulling, the raw flour was defatted using 90% ethanol. The defatting process involved mixing ethanol and flour at 1:10 (flour to ethanol) for 12 h. After 12 h, the supernatant was decanted, and the residue dried in a fume hood as defatted flour.

Similarly, the egusi seeds were sorted to remove chaff and other extraneous materials. They were then milled and defatted using a cold-press method (One Market Automatic Oil Press Machine, Double-lion Grain & Oil Machinery Co., Ltd., Zhengzhou, China).

**Figure 1:**
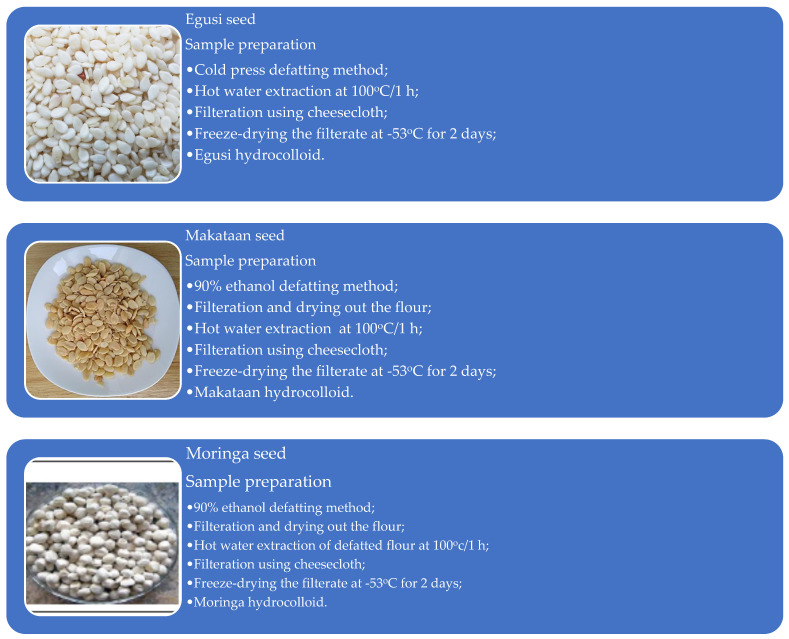
Seed preparation and extraction of moringa, makataan, and egusi hydrocolloids.

MOLS were sorted, dehulled, and milled using a hammer mill. The resulting flour was defatted continuously with 90% ethanol at a ratio of 1:10 (flour to ethanol) for 12 h at room temperature and stirred using a magnetic stirrer (Worldofscience Advanced 340 °C Hotplate including E-PT1000-23, 20 Litre Magnetic Stirrer, LCD Display with RS232 interface, Cape Town, South Africa). The mixture of flour and ethanol was processed according to the methods used for makataan, as shown in Figure 1. The defatted dried flours were packed in Ziplock bags and stored in the refrigerator at 4 °C until further use.

### 2.3. Hot Water Extraction of Moringa, Makataan, and Egusi Hydrocolloids

Using the modified method of Olubi [7,14], defatted moringa, egusi, and makataan seed flours were suspended in purified water (1:25 *w/v* flour: water ratio), and the suspension was cooked for an hour at 100 °C on an electric stove [7,15]. The residue was filtered through several layers of cheesecloth. The procedure was performed twice, while the defatted flour was suspended in boiling water for a second time. Following its combination, the filtrate was placed in a freezer (−50 °C) (Eppendorf F740020016 Freezers CryoCube (740 L, −86 to −50 °C, Hanoi, Vietnam)) to maintain the sample’s frozen state for later freeze-drying (United Scientific CryoDry Level 12, 37 York Street, Sydney, NSW 2000, Australia). The moringa seed hydrocolloid (MOSH), egusi seed hydrocolloid (EGSH), and makataan seed hydrocolloid (MASH) were recovered after a 48 h freeze-drying process at −53 to +55 °C and ±150 Torr of pressure. The freeze-dried samples were refrigerated until further analysis. The EGSH, MOSH, and makataan MASH hydrocolloids were analysed for their proximate amino acids and sugar content. The functional analysis entails colour, scanning electron microscopy, and light microscopy to observe the morphology of the hydrocolloids.

### 2.4. Proximate Analysis of Moringa, Makataan, and Egusi Hydrocolloids

The moisture content and dry matter in the hydrocolloid samples were determined using the AOAC Method 934.01 [16]. After burning in a muffle furnace (Laboratory 1200C 64L Heating Electric Muffle Furnace With K Type Thermocouple, Xiamen, China), the amount of ash was measured gravimetrically (according to AOAC Method 942.05, for two hours at 900 °C [16]. The amount of protein was calculated as a percentage of nitrogen determined using a nitrogen analyser (Leco Truspe N-630-100-200-230 V Amps 12 A, Saint Joseph, MI, USA) [16]. Total carbohydrate was calculated by the difference.

### 2.5. Microstructural Analysis of Moringa, Makataan, and Egusi Seed Hydrocolloids Using Scanning Electron Microscopy (SEM)

The MOSH, EGSH, and MASH samples were dried in a 50 °C oven and then placed in a glass desiccator containing anhydrous silica gel for the night before being subjected to SEM examinations. Before applying a gold coating using a Bal-tec SCD 005 sputter coater (Bal-tec AG, Balzers, Liechtenstein), the triplicate hydrocolloid samples were mounted onto an SEM specimen stub using double-sided carbon tape. A Phillips ×L 30 SEM (SEMTech Solutions, Inc., Billerica, MA, USA) was then used to take pictures of the specimens at a magnification of 250 [17].

### 2.6. Colour Parameters of Moringa, Makataan, and Egusi Hydrocolloids

According to the procedure described by Jakopic et al., the spectrophotometer (Vis Spectrophotometer vinma × 721 LDC Digital Lab Visible Spectrophotometer 350–1020 nm Lamp Lab Constantia, Kloof) was used to measure the colour of EGSH, MASH, and MOSH [18]. Following system calibration, the following parameters were found: a* (red-green component: −a* = greenness and + a* = redness), b* (yellow-blue component: −b* = blueness and + b* = yellowness), and L* (luminosity or brightness: L* = 0 black and L* = 100 white). Every analysis was carried out three times. Potato starch was utilised as a benchmark for comparative analysis. Equation (1) was used to compute the hue angle, and Equation (2) for the chroma (C*).

(1) $$\mathrm{Hue}\;\mathrm{angle}=\tan^{-1}\;(\frac{b}{a})$$

(2) $$C^{*}=\sqrt{{\left(a*\right)}^{2}+({b*)}^{2}}$$

where b* = yellow-blue component, and b* = yellow-blue component.

### 2.7. Light and Polarised Light Microscopy of Moringa, Makataan, and Egusi Hydrocolloids

The MOSH, EGSH, and MASH were each tested in triplicate on a microscope slide, combined with a drop of distilled water and secured with a coverslip. Using an optical microscope (Leica Microsystems, Wetzlar, Germany) with a digital camera at a magnification of ×40, the hydrocolloid granules were observed in both polarised and unpolarised modes (Lexica) [19].

### 2.8. Particle Size Determination of Moringa, Makataan, and Egusi Hydrocolloids

To assess the size distribution of dispersed particles, we employed the Mastersizer 2000 instrument (Malvern/microcell/Claisse/PANanalytical Products, Enigma Business Park, Grovewood Road, WR14 1×Z, Malvern, UK) which uses laser diffractography. The measurement procedure was based on sample dispersion controlled by software and the angle dependence of the scattering intensity of a collimated He-Ne laser beam. The hydrocolloid powders were mixed with deionised water at 2000 rpm for two minutes, with a refractive index of 1.36 for both the water and the hydrocolloid. The median diameter of the granules, which is the diameter of 50% of the particles by volume, was used to express the particle size [20].

### 2.9. Interfacial Tension of Moringa, Makataan, and Egusi Hydrocolloids

**Figure 2:**
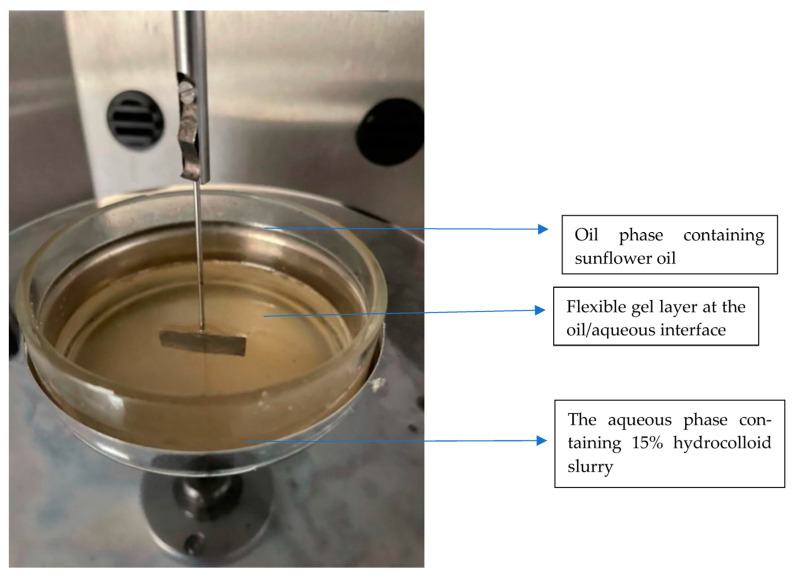
Hydrocolloid layer formation across the sunflower interface.

Using the Kruss K100 tensiometer (85 Borsteler Chaussee, Hamburg, Germany), interfacial tension measurements were carried out using the Wilhelmy plate method as the basis for the measurements. Approximately 15 mL of each hydrocolloid slurry was prepared after mixing 10 g of hydrocolloid with 10 g of water at room temperature. The mixture was poured into a spotless glass dish with a 70 mm diameter. Next, a hydrophilic platinum plate was suspended vertically from a force transducer sensitive enough to penetrate the surface by the plate’s lower edge. Before completely submerging the plate, around 50 mL of the sunflower oil-based oil phase was introduced, as seen in Figure 2. Every measurement was calibrated at 7200 s to enable the system to achieve an accurate measurement [21].

### 2.10. Visual Appearance

The hydrocolloids were prepared at a ratio of 1:1 (hydrocolloid to water) to investigate the visual appearance of egusi, makataan, and moringa hydrocolloids. Samples were taken at Day 0 and after 10 days of storage under controlled conditions of 40 °C in the presence of light. The stability and colour changes in the hydrocolloids were evaluated and recorded at 24-h intervals, following a methodology similar to that of de Almeida Paula et al. [22], with slight modifications.

### 2.11. Amino Acid Composition of Moringa, Makataan, and Egusi Seed Hydrocolloids

The modified ninhydrin method was used as described by Li et al. [23]. Ninhydrin (2 g) was dissolved in distilled water (100 mL) under a stream of nitrogen gas to prepare a ninhydrin solution. Ninhydrin solution (1 mL) was added to 0.1% (wt) aqueous sample solution, heated in a boiling water bath for 5 min, and immediately cooled in an ice bath. Cooling is essential to stop the reaction and stabilise colour development. After 5 min of heating, the reaction vial was immediately transferred to an ice bath to cool the solution rapidly. The absorbance measurement measured the absorbance of each hydrocolloid sample at 580 nm using an Agilent 8453 spectrophotometer. Distilled water was used as a blank for baseline correction with an Agilent 8453 spectrophotometer in Santa Clara, California (USA). The absorbance for each sample was detected with a calibration curve or standard amino acid solutions to relate absorbance to amino acid concentration. A constant calibration check was performed to verify the spectrophotometer’s performance.

### 2.12. Sugar Profiling of Moringa, Makataan, and Egusi Seed Hydrocolloids

Wang et al. [24] report that a mixture of monosaccharide standards was used to prepare the solution. Hydrocolloids dissolved more easily in deionised water when the concentration was 3 mg/mL. The hydrocolloid in 10 mL of solution was then treated with 10 mL of 4 M trifluoroacetic acid for two hours at 100 °C to hydrolyse it to its monomers. It was underlined that the hydrolysis phase came before the derivatisation of 1-phenyl-3-methyl-5-pyrazolone.

An Agilent Technologies ZORBA× Eclipse Plus-C18 HPLC column (250 mm length, 4.6 mm internal diameter, and 5 μm particle size) was used to separate the monosaccharides. The water 2489 UV/V detector-equipped Waters e2695 HPLC was employed.

### 2.13. Statistical Analysis

The results were presented as the mean ± standard deviation of triplicate measurements. Multivariate analysis of variance was conducted to determine the mean differences between treatments where significant (*p* ≤ 0.05) differences existed. Duncan’s multiple range test was employed to separate the means. All data analysis was conducted using the IBM SPSS version 29 software (2022).

## 3. Results and Discussion

### 3.1. Hydrocolloid Extraction Yield from Hot Water Extraction

**Figure 3:**
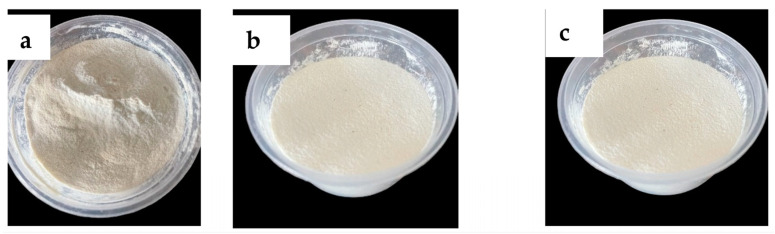
Hydrocolloids from (**a**) moringa seed, (**b**) egusi seed, and (**c**) makataan seed.

**Figure 4:**
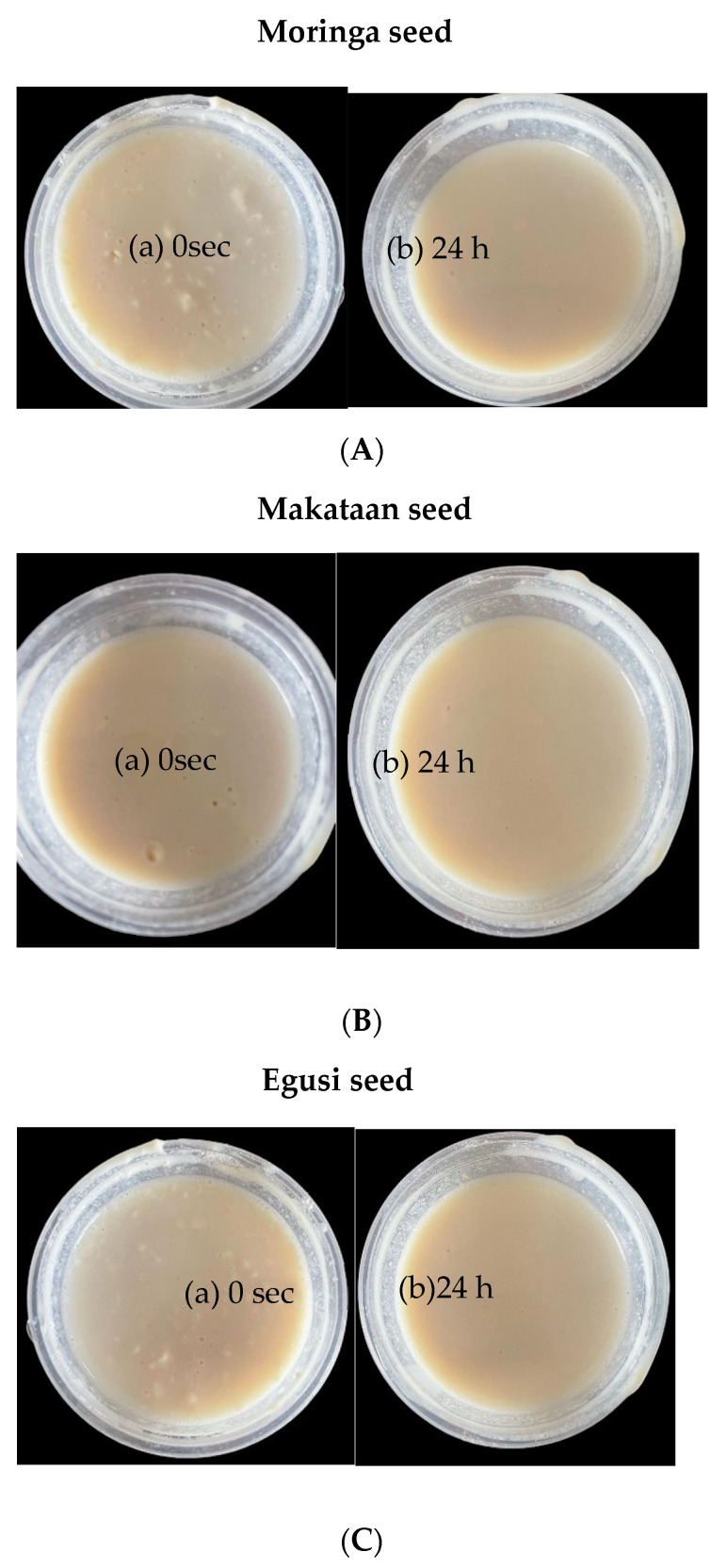
Visuals of room-temperature water to hydrocolloid (10:10) mix of (**A**) moringa seed (a) 0 s, (b) 24 h; (**B**) makataan seed (a) 0 s, (b) 24 h; and (**C**) (a) 0 s, (b) 24 h egusi seed hydrocolloid.

The yield of makataan, egusi, and moringa seed hydrocolloids (MASH, EGSH, and MOSH, respectively) and their physical and textural properties differed significantly among the samples. The images of each hydrocolloid show a creamy white powdery flour, as seen in Figure 3. The hydrocolloids of EGSH and MASH gave a 52% yield, while the yield of MOSH was only 42%. It is worth mentioning that some proteins and carbohydrates, which had the highest composition, were dissolved, as seen from the brown slurry images in Figure 4. This finding is similar to the review reported by Hedayati et al. [25], who found that the mucilage seeds were not damaged and the ultrasonically extracted mucilage powder was purer.

### 3.2. Proximate Composition of Moringa, Makataan, and Egusi Hydrocolloids

The proximate compositions of egusi seed hydrocolloid, makataan seed hydrocolloid, and moringa seed hydrocolloid, respectively, EGSH, MASH, and MOSH, alongside their raw and defatted flour are presented in Table 1. The moisture content of the hydrocolloids varied from 7.01 to 7.80% (*w*/*w* dry weight). The moisture of the raw undefatted flour from the three oilseeds was higher than the defatted flour and was significantly (*p* ≤ 0.05) different. The moisture content of the undefatted flours from egusi, makataan, and moringa seeds ranged from 11.34 to 12.96% (*w*/*w* dry weight). These values were higher than the moisture content reported in previous studies on five other species of cucurbit seed (6.45% to 7.56%) by Dimitry et al. [26]. However, they were lower than the moisture content found in raw Ukpo (*Mucuna flagellopes*) seeds (approximately 15.50%), as reported by Okorie et al. [27].

The moisture content of the defatted oilseed flours ranged from 7.54 to 10.73%. These values were higher than the moisture content obtained from defatted Guna flour (5.50%) [28]. The raw egusi exhibited the highest moisture content among all the samples.

The protein content of the oilseed hydrocolloids was higher than that of the raw oilseeds, with a percentage of 48.12, 34.00, and 35.00% for MASH, MOSH, and EGSH, respectively. Interestingly, the protein content of the defatted oilseeds conspicuously increased to 39.07%, 40.88%, and 58.18% for defatted makataan seed flour, defatted moringa seed flour, and defatted seed egusi flour (DMASF, DMOSF, and DESF) respectively, compared to the raw oilseeds and hydrocolloids. Notwithstanding, there was an exception for MASH (39.07%), which had a higher protein content than the raw oilseed (27.73%). The increased protein content of the defatted flour from the three oilseeds had a reduced carbohydrate content, as observed with the raw oilseeds, although the carbohydrate in raw flour was higher. The low carbohydrate content (Table 1) might be the reason for the comparatively high amount of protein in the three raw oilseeds and the defatted oilseed flours. Food gums with high protein are known to be natural stabilisers. Emulsions form the basis of a vast range of food products, where those stabilised by proteins are of great interest [29]. It is worth mentioning that the protein content obtained in this study was similar and within the range of some other previously reported studies [7,30,31,32,33].

The interfacial properties of proteins have been studied extensively in food colloid research. The emulsifying properties of proteins depend on two effects: (1) a substantial decrease in the interfacial tension due to the adsorption of the protein at the oil–water interface; and (2) the electrostatic, structural, and mechanical energy barrier caused by the interfacial layer that opposes the destabilisation processes [34,35]. The increase in macronutrients shows that the defatting methods used for the sample preparation effectively produced nutritious flour, similar to the report by Famakinwa et al. [36], who reported increased antioxidant activity and nutrients of wheat flour after defatting. Thus, the protein in hydrocolloids isolated from all three sources (Table 2) might be precipitated at pH 4.0–4.5 due to the isoelectric point falling in that range. The protein content of EGSH significantly (*p* ≤ 0.05) differed from MOSH and MASH. It is well documented that protein plays an essential role in the emulsifying activities of naturally occurring emulsifiers, such as gum arabic. Proteins emulsify a polysaccharide covalently attached with a glycosidic linkage [36,37,38]. In the case of gum arabic, the covalently attached proteins provide additional functionality to the polysaccharide. The presence of proteins enhances the emulsifying properties of the gum arabic by improving its ability to form and stabilise oil-in-water emulsions. The proteins act as emulsifiers, promoting a stable interfacial film between the oil and water phases. This film prevents the coalescence and separation of the dispersed oil droplets, thus increasing the emulsion stability.

### 3.3. Sugar Composition of Egusi, Makataan, and Moringa Seed Hydrocolloid

The sugar composition of MASH, EGSH, and MOSH are presented in Table 3. The hydrocolloids from all three sources have predominant monomeric sugar units: glucose, a trace amount of fructose (0.35 to 0.57 g/100 g); a dimeric unit: sucrose; a trimeric unit: raffinose; and a tetrasaccharide unit: stachyose. Sucrose combines two monosaccharides (glucose and fructose) joined together as disaccharides. Sucrose is found mainly in plants and can be crystallised into refined sugar. The three hydrocolloids showed a high sucrose and raffinose content ranging from 4.95 to 7.68 g/100 g and 0.48 to 5.99 g/100 g, respectively. During storage, amorphous sucrose tends to crystallise to the more thermodynamically stable crystalline form [31]. Raffinose is a trisaccharide composed of galactose, glucose, and fructose. It is found in beans, cabbage, Brussels sprouts, broccoli, asparagus, other vegetables, and whole grains. MASH and EGSH are high in raffinose, 5.85 and 5.99 g/100 g, respectively, compared to MOSH (0.48 g/100 g), while MOSH is higher in sucrose (7.68 g/100 g) than MASH and EGSH (4.95 and 5.90 g/100 g, respectively). The raffinose family of oligosaccharides are α-1, 6-galactosyl extensions of sucrose [39] found in plants and known to serve as a seed desiccation protectant. They transport sugar in phloem sap and can sometimes act as non-digestible human storage sugars [40]. These oligosaccharides pass undigested through the stomach and small intestine. In the large intestine, they are fermented by bacteria that possess the α-GAL enzyme and produce short-chain fatty acids (SCFA) (acetic, propionic, butyric acids) and the flatulence commonly associated with eating beans and other vegetables [24].

Before freezing procedures (cryopreservation), raffinose provides hypertonicity for cell desiccation. Raffinose or sucrose is used as a base substance for sucralose [41]. Raffinose is also employed in skin moisturisers, smoothers, and prebiotics. It allegedly promotes the growth of lactobacilli and bifidobacterial in food or drinks. The low level of glucose in MASH and EGSH (0.49 and 0.78 g/100 g, respectively) could be due to high sucrose from all three oilseed sources (4.95 and 5.90 g/100 g). The three hydrocolloids also contain stachyose ranging from 0.38 to 1.48 g/100 g. Stachyose is one of the most abundant tetrasaccharides in plants, and it is a higher homolog of raffinose (Table 3).

First isolated from the rhizomes of *Stachys tuberifera*, stachyose coexists with raffinose and other related oligosaccharides in various organs of many plant species [42]. Reports have it that stachyose inhibits cancer cell proliferation and conversely promotes probiotics’ proliferation, which induces their apoptosis in a dose-dependent manner, thereby preventing colon cancer indirectly [43]. Additionally, sucrose and fructose can help to preserve food by inhibiting microbial growth and extending shelf life. These sugars promote browning reactions and flavour development in specific processes, such as baking or caramelisation. Furthermore, fructose may provide antioxidant properties, contributing to potential health benefits. 

In conclusion, these hydrocolloids with high sucrose and fructose content offer several benefits in various food applications. These benefits include their role as natural sweeteners, providing a pleasant taste and flavour enhancement. The high sugar content can serve as a readily available energy source and contribute to the texture of food products, improving mouthfeel and overall eating experience. However, consuming hydrocolloids with high sucrose and fructose content in moderation is essential, as excessive consumption of these sugars, particularly in processed and high-calorie foods, can have adverse health effects. Balancing their intake as part of a well-rounded diet is crucial to ensure optimal health and well-being.

### 3.4. Amino Acids in Makataan, Moringa, and Egusi Seed Hydrocolloids

The amino acid compositions of the three oilseed hydrocolloids are shown in Table 4. Each hydrocolloid contained all essential amino acids: threonine, phenylalanine, valine, methionine, lysine, histidine, and leucine, although with significant (*p* ≤ 0.05) variation in content. MASH was characterised by high glutamine, arginine, aspartine, and glycine. Wang et al. [44] reported that after grafting with the amino acids, the viscosity of the amidated pectin was significantly improved. Each hydrocolloid contained appreciable quantities of valine (0.75 to 1.30 g/100 g) and histidine (0.42 to 0.92) mg/100 g) (MOSH). The methionine ranged from 0.75 to 1.58 g/100 g, with the highest in EGSH. The ability of a protein to possess suitable emulsifying and foaming properties depends mainly on the protein conformation and its ability to interact with water and oil at a molecular level. Amino acids are grouped according to their side chains. The nine amino acids with *hydrophobic* side chains are glycine (Gly), alanine (Ala), valine (Val), leucine (Leu), isoleucine (Ile), proline (Pro), phenylalanine (Phe), methionine (Met), and tryptophan (Trp). The three hydrocolloids contain all the hydrophobic amino acids except tryptophan. The side chains are composed chiefly of carbon and hydrogen, have tiny dipole moments, and tend to repel water. There are six polar amino acids with no charged side chains. These are serine (Ser), threonine (Thr), cysteine (Cys), asparagine (Asn), glutamine (Gln), and tyrosine (Tyr). Four polar amino acids are present in the three hydrocolloid samples. These amino acids are usually found at the surface of proteins [45]. Among the various classes of amino acids, depending on the propensity of the side chain to interact with a water molecule, the three hydrocolloids possessed the highest content of hydrophobic amino acids compared to soy lecithin, followed by neutral, basic, and acidic amino acids, and this was a similar trend to that recorded in another study [24]. It is worth noting that the hydrophobic protein content of the three hydrocolloids follows the trend [46]: MOSH > EGSH = MASH. It is worth mentioning that the specific amino acid profile and content varies among different hydrocolloid sources. Additionally, hydrocolloids’ overall nutritional impact and health benefits depend on various factors, including processing methods, product formulation, and individual dietary needs. Hydrocolloids with high amino acid content can serve as a valuable protein source for individuals following vegan or vegetarian diets, where obtaining sufficient protein can be challenging. These hydrocolloids can provide essential amino acids to meet protein requirements [47].

### 3.5. Functional Properties of Moringa, Egusi, and Makataan Seed Hydrocolloid

#### Visual Appearance and Particle Size Distribution of Egusi, Makataan, and Moringa Seed Hydrocolloids

**Figure 5:**
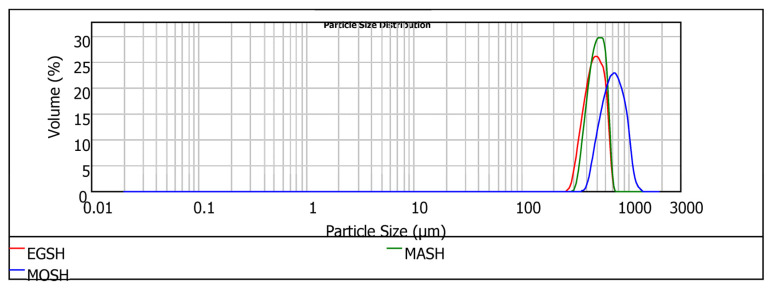
Histogram of the particle size distribution of the milled hydrocolloids of egusi (EGSH), makataan (MASH), and moringa (MOSH).

When milled into different fractions, the hydrocolloids experienced mechanical and thermal energy, resulting in multiple particle sizes and distributions, as shown in Figure 5 and Table 5. 

**Figure 6:**
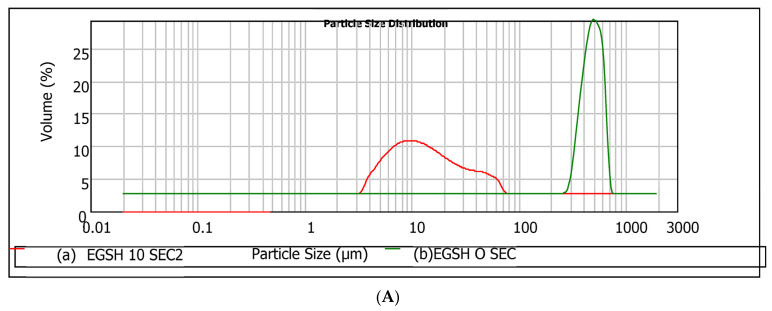

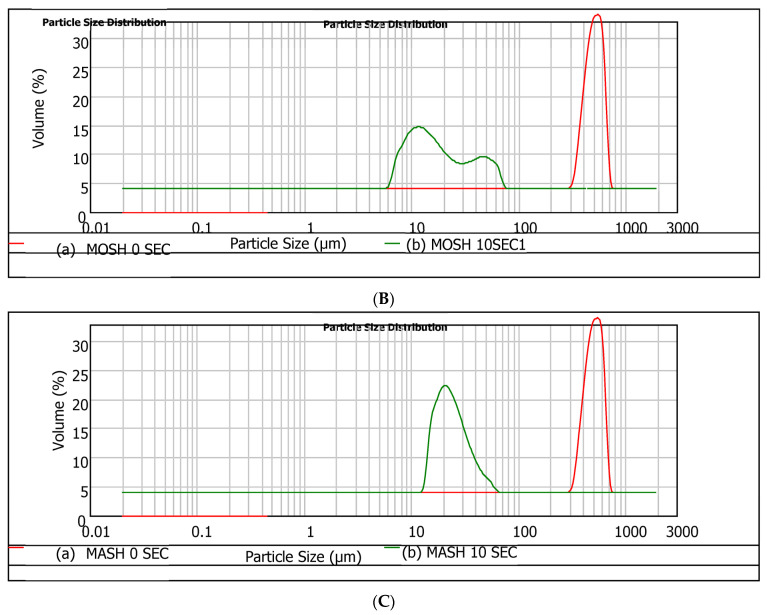
Histogram of changes in the particle size distribution of hydrocolloids after 10 s: (**A**) egusi seed hydrocolloid; (**B**) moringa seed hydrocolloid; (**C**) makataan seed hydrocolloids.

Regardless of the hydrocolloid type, particles from the milling process consisted of relatively large aggregates. The high particle size of the three hydrocolloids indicates that they will offer a more robust particle in solution, enhancing the absorption dissolution rate in solution. The particle size (D (0.5)) follows the trend MOSH > MASH > EGSH, whereas the span shows a different trend: MOSH > EGSH > MASH. Even though visual observation revealed that the physical appearances of EGSH and MASH were quite close (larger aggregates of particles had rough surfaces than the fine fraction), EGSH hydrocolloids gave rise to the smallest size. MASH demonstrated a more uniform distribution of particles. Moreover, visual observation of egusi and makataan hydrocolloids revealed a high adhesion tendency of their particles, which could be due to the aggregation of the protein-based particles leading to increased water absorption and homogeneity within the film structure. Conversely to EGSH and MASH, MOSH comprised large numbers of smoothly disintegrated larger particle sizes (756.44 µm) and these were less evenly distributed (0.20). This particle size distribution is directly related to the higher mechanical performance of the hydrocolloid from moringa seeds compared to EGSH and MASH. A similar morphology was observed by Lin et al. [48] in a soya lecithin substitute for eggs, which presented a significant concentration of stable aggregates. Bearing in mind that hydrocolloids (proteins and polysaccharides) are soluble in water, resulting in a reduction in their size [49], the droplet size measurement was then taken every two seconds until the process showed no significant changes in the sizes of particles. This observation interval is relevant for the rheological characterisation of these systems. The required time to reach the equilibrium particle size was 10 s in our experimental conditions. The main results of particle size and particle size distributions after 10 s are presented in Table 6 and Figure 6, respectively. Outcomes associated with the initial time (t = 0 s) are compared.

Based on the histograms and Table 6, regardless of the hydrocolloid type, the equilibrium particle size after 10 s reached a significantly low value, suggesting a high dissolution rate. This enhancement in the rate is partly related to the agitation of the system (2000 rpm) during droplet size measurement [50]. The trend EGSH < MOSH < MASH suggests that despite the higher specific areas of MASH particles (small particle size with higher to MOSH), these particles are more difficult to dissolve than MOSH. In conclusion, the trend observed with EGSH < MOSH < MASH, regarding solubility, indicated that despite the smaller particle size and higher roughness of MASH particles compared to MOSH, it was more challenging to dissolve. This finding has implications for the interfacial behaviour of the hydrocolloids and highlights an additional distinction between MASH and EGSH despite belonging to the same botanical family.

### 3.6. Microstructure of Moringa, Makataan, and Egusi Seed Hydrocolloids

**Figure 7:**
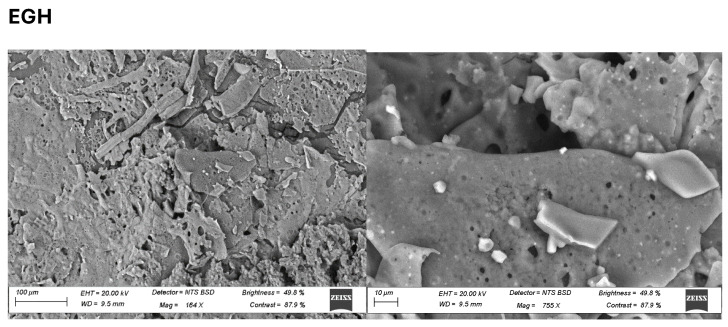

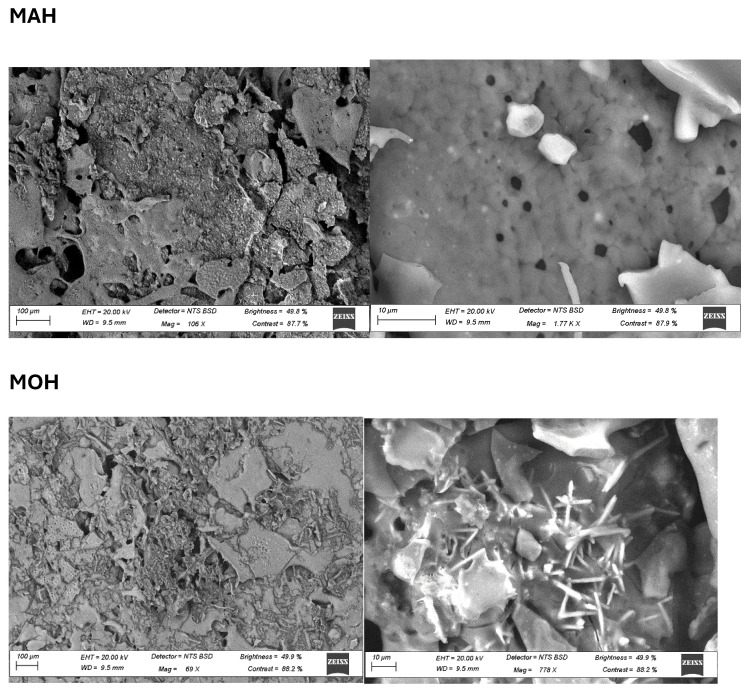
Scanning electron microscopy (SEM) images of freeze-dried moringa seed hydrocolloid (MOSH), makataan seed hydrocolloid (MASH), and egusi seed hydrocolloid (EGSH).

The microstructure of the hydrocolloids differed considerably with each hydrocolloid. The MOSH showed a fibrous microstructure composed of loose, interconnected fibres with small cavities [51]. Fewer fibres were evident than in MASH and EGSH. Haiyan et al. [52] also observed a low-density network of agar gels with tiny voids, thus reflecting fewer junction zones between the propellers. Highly interconnected networks are formed in MASH (Figure 7), creating a highly dense and compact structure. The lower the concentration/volume of the hydrocolloid in the solution, the thinner and the more dispersed the molecular bundles, decreasing the gelled system’s springiness and hardness. The compact microstructure observed in MASH and EGSH was lower in springiness compared to MOSH, similar to Tiwari et al.’s study [53]. Yuris et al. report that the microstructures of gels obtained with mango pulp, gum gellan, and agar showed a better uniformity of cellular structures, where the air cells were homogeneous and of a reduced size. According to Yuris et al., MOSH showed a more homogeneous network with smaller polymer-free cavities than the others (Figure 7) [54]; larger pores indicate the presence of water in large quantities inside the gel network, probably due to the absence of a homogeneous network with large cavities.

Aeration during freezing and freeze-drying produced highly uniform samples, indicating that mass and heat transfer were enough to avoid trapped vapour. Different microstructures were identified in MOSH, MASH, and EGSH. The MASH and EGSH presented a denser, more compact, and more robust network, leading to high retention, while the MOSH showed a weaker network. Additionally, the elemental composition of each hydrocolloid was observed with the EDX analyses, indicating that carbon and oxygen peaked as the highest among all the elements in the three hydrocolloids, as seen in Table 7. The micrographs and the EDX spectrum revealed that the homogeneous distribution of the hydrocolloid particles over the surface (Figure 7) might serve as an artificial bone-like network for cells in vitro. Carbon, oxygen, sulphur, phosphorus, potassium, and calcium were visibly peaked and varied amongst the hydrocolloids. MOSH has the highest sulphur and potassium compared to EGSH and MOSH.

### 3.7. Microscopy Light and Partially Polarised Light of Egusi, Moringa, and Makataan Seed Hydrocolloid

**Figure 8:**
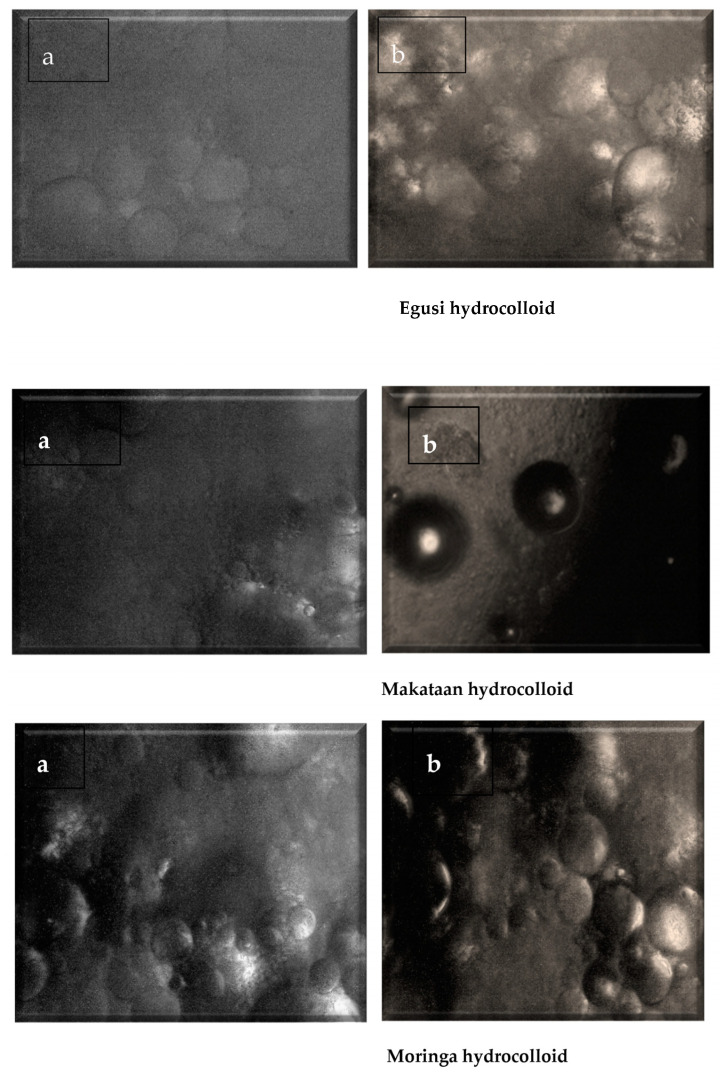
Morphology of extracted hydrocolloid using light and polarised microscopy: (**a**) light, (**b**) partially polarised.

Particle size reduction observed at a magnification of ×40 also confirmed that grinding and sieving effectively reduced the particle size of each hydrocolloid. Morphological results of each hydrocolloid using polarised and non-polarised light are shown in Figure 8. Each hydrocolloid was a semi-crystalline polymer alternately stacked with semi-crystalline and vague growth rings. The primary components were microprotein, linear amylose polymers, and highly branching, partially crystalline amylopectin. Under a polarising microscope, the starch particles displayed birefringence and cross-polarized crosses.

### 3.8. Colour of Moringa, Egusi, and Makataan Hydrocolloids

For each hydrocolloid, the hue was 87.99, 87.03, and 86.87 for EGSH, MOSH, and MASH, with no significant differences. Chroma is the attribute that expresses the purity of a colour. The chroma for each hydrocolloid, 16.12, 17.65, and 16.50, for EGSH, MOSH, and MASH, respectively, indicated less colour saturation. The lightness (L*), greenness (a*), and redness (b*) of each hydrocolloid significantly varied by 83.03, 0.54, and 16.12; 81.39, 0.61, and 17.65; and 78.63, 0.90, and 16.50, respectively, for egusi seed hydrocolloid (EGSH), moringa seed hydrocolloid (MOSH), and makataan seed hydrocolloid (MASH). MASH showed the lowest lightness, 78.63, with redness (a*) close to zero for all hydrocolloids (0.54 to 0.90), implying a neutral colour.

Furthermore, the yellowness (b*) differed significantly from 16.12 to 17.65, being more yellowish than blue, as shown in Table 8. The oilseed sources played a significant role in the colour of each hydrocolloid. The lightness-defining colour in terms of how close it is to white or black is a property of the seed coat’s type and the number of pigments (tannins and anthocyanins). It depends primarily on plant genetics. The exposure of cotyledons after grinding seeds may be the possible reason for differences in colour parameters amongst the hydrocolloids. The light colour of all the hydrocolloids may find specific applications in foods that require little or no colour change during processing. 

### 3.9. Principal Component of Egusi, Makataan, and Moringa Seed Hydrocolloids

**Figure 9:**
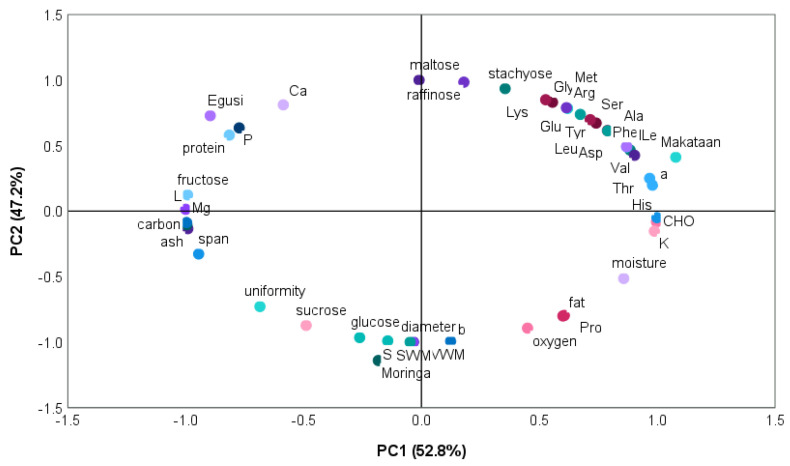
Principal component scores and loading plots for the nutritional and functional relationship among the hydrocolloids.

Figure 9 illustrates how two factors contributed to the variance in the nutritional and functional characteristics of the hydrocolloids from egusi, makataan, and moringa. Principal Component 1 (PC1) accounted for 52.8% of the variability, whereas Principal Component 2 (PC2) accounted for 47.2%. PC1 was the most significant source of variability. The two components’ combined variability added up to 99%.

PC1 related to makataan hydrocolloid (MASH) positively correlated with amino acids (ala, phy, asp, val, ile, thr, his, pro), carbohydrate, potassium, and fat. It negatively correlated with some sugars (sucrose, glucose) and related to moringa seed hydrocolloid (MOSH). MASH possessed a combination of functionalities, increased hydrophobicity, water binding, potential charge interaction, and diverse functionalities, including carbohydrates and fat. Sucrose and glucose in MOSH could contribute to thickening properties as the sugars interact with other components or concentrate in processing. 

PC2 related to egusi seed hydrocolloid (EGSH) positively correlated with amino acids (tyr, ser, glu, arg, lys, gly, met), stachyose, calcium, phosphorus, and protein. EGSH possessed a combination of functionalities, moderate hydrophobicity, water binding, and potential charge effects interacting with other charged molecules influencing thickening or gelling behaviour potentially modulated by calcium and nutritional contribution due to high protein and stachyose. 

The three hydrocolloids also behaved differently, with MOSH having lesser nutritional and functional qualities than makataan and egusi hydrocolloids. 

### 3.10. Interfacial Tension of Egusi, Moringa, and Makataan Hydrocolloids

**Figure 10:**
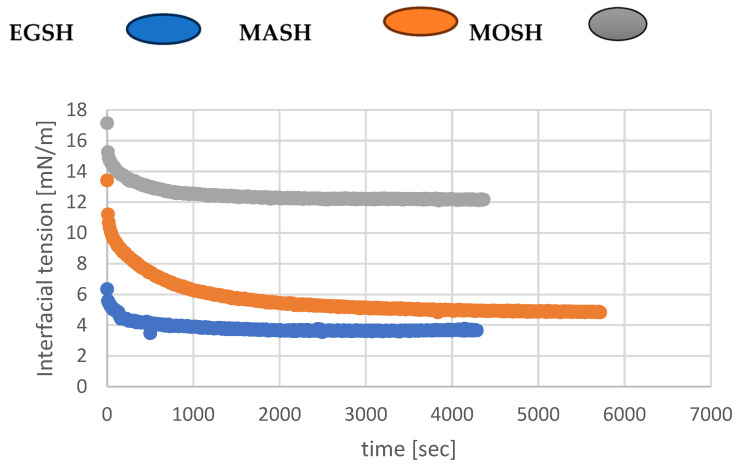
Dynamic interfacial tensions of the three hydrocolloids.

The previous sections made it clear that dispersion of the three hydrocolloids (EGSH, MASH, MOSH) in water gave rise to a system containing solid particles in mixtures with an aqueous solution of proteins and polysaccharides, which is the result of hydrocolloid dissolution. The primary purpose of this part of the present investigation was to determine the extent to which the mixed emulsifier system formed by solid particles (hydrocolloids) and mainly their proteins can modify oil–water interfacial properties. The dynamic interfacial tensions of the three systems were first measured to probe the reduction rate of the interfacial tension and shed light on the mechanism of the emulsion preparation. Then, the equilibrium interfacial tensions were extracted from the graphs to estimate the emulsion stabilisation mechanisms against coalescence and creaming. The results of the dynamic interfacial tension are shown in Figure 10.

Figure 10 clearly shows the reduction rate of the interfacial tension, as measured by the slopes of the curves shown for each hydrocolloid. This is in line with the particle dissolution rate in water to release the surface active species (proteins) (see Section 3.5) [55]. Additionally, all hydrocolloids reached the equilibrium interfacial tension; the values are summarised in Table 9. Data related to some conventional surfactants, that is, lecithin and guar, are also shown for comparison [56].

Conversely to MOSH, which has an equilibrium interfacial tension higher than the ones associated with conventional surfactants (guar, lecithin), EGSH and MASH demonstrated excellent interfacial activities. The equilibrium interfacial tension of EGSH and MASH is lower than the one associated with lecithin and comparable to the one related to guar [57]. This is deemed a critical finding for both scientific interest and technological applications of these systems. Protein plays an essential role in the emulsifying activities of naturally occurring emulsifiers, such as gum arabic [58]. Attaining this perfect extraction state is only possible by using an environmentally friendly process that uses heat instead of the denaturing effect of using a solvent extraction method to obtain hydrocolloids [59].

Additionally, the ability of a protein to possess suitable emulsifying and foaming properties depends mainly on the protein conformation and its ability to interact with water and oil at a molecular level. The rate of interfacial tension reduction in the presence of MASH is low (despite its highest protein content) compared to the ones associated with EGSH and MOSH which could be due, first, to the slow rate of proteins released from the hydrocolloids. Second, this effect could be due the to slow interfacial rearrangements of its proteins over time to adopt the equilibrium conformation where polar segments move from the oil phase; only non-polar segments adsorb at the interface [60]. This agrees with the high content of amino acids with polar and non-polar side chains in MASH compared to MOSH or EGSH. Third, MASH particles could take longer before reaching a size small enough to be adsorbed at the interface.

On the other hand, it seems reasonable to assume that MOSH’s high equilibrium interfacial tension, compared to MASH or EGSH, is caused by its high hydrophobic protein content (hydrophobic proteins MASH = hydrophobic proteins EGSH > hydrophobic proteins MOSH). Proteins in MOSH likely have more attachment points than the ones in MASH or EGSH, reducing the amount of MOSH adsorbed as, in this case, each protein could occupy more sites than MASH or EGSH.

### 3.11. Conclusions and Recommendation

This study explored the potential of EGSH, MASH, and MOSH as natural emulsifiers that enhance emulsion stability. The hydrocolloids, extracted from egusi, moringa, and makataan seeds through a hot water extraction process at 100 °C with a 1:8 ratio, were characterised by their physicochemical and functional properties. The hydrocolloids possessed a higher protein-to-carbohydrate ratio, contributing to improved food quality, enhanced nutritional properties, and heightened interfacial activity. The hydrocolloid/dissolved protein system could anchor onto the interface, resulting in equilibrium interfacial tensions comparable to those achieved with standard surfactants. The superior performance exhibited by EGSH and MASH in this content is noteworthy. Additionally, EGSH and MASH hydrocolloids showcased robust particle adhesion, likely attributed to the substantial size of protein-based particles and their consequent high-water absorption, leading to enhanced structural homogeneity. These findings suggest that emulsifier systems comprising hydrocolloids with a high protein content hold promise for food product development. Such systems offer manufacturers greater flexibility in selecting hydrocolloid/dissolved protein mixtures to control rheology—an imperative property for the technological applications of these systems. However, further research is necessary to comprehensively understand and optimise these hydrocolloids’ performance, considering the limitations and advantages uncovered in this study in the broader context of food industry applications.

## Figures and Tables

**Table 1:** Proximate composition of raw, defatted hydrocolloid of the makataan, moringa, and egusi oilseeds ^1,2^.

	Raw Flour			Hydrocolloid			Defatted Flour		
Proximate % *w*/*w* Dry Weight	RMAS	RMOS	REFS	MOSH	EGSH	DMASF	DMOSF	MASH	DESF
Protein	27.73 ± 1.37 ^a^	30.08 ± 0.88 ^b^	32.00 ± 3.00 ^c^	34.00 ± 2.65 ^d^	35.00 ± 1.00 ^d^	39.07 ± 0.31 ^e^	40.88 ±1.09 ^e^	48.12 ±1.00 ^f^	58.12 ± 1.06 ^g^
Moisture	12.44 ± 0.85 ^a^	11.34 ± 0.55 ^b^	12.96 ± 0.69 ^a^	7.80 ± 0.92 ^c^	7.01 ± 0.28 ^c^	8.01 ± 0.01 ^c^	7.54 ± 0.16 ^c^	7.11 ± 1.04 ^a^	10.73 ± 0.55 ^b^
Ash	3.63 ± 0.56 ^a^	5.09 ± 0.48 ^a^	3.39 ± 0.72 ^a^	4.09 ± 3.26 ^a^	5.98 ± 0.05 ^b^	12.67 ± 0.58 ^d^	10.76 ± 1.26 ^d^	6.52 ± 1.48 ^c^	15.23 ± 0.48 ^e^
Fat	36.12 ± 1.11 ^a^	38.26 ± 0.65 ^b^	37.73 ± 4.44 ^b^	4.53 ± 0.42 ^c^	5.88 ± 0.42 ^d^	4.68 ± 0.56 ^c^	5.52 ± 0.23 ^d^	0.53 ± 0.06 ^a^	1.54 ± 0.40 ^e^
Carbohydrates	10.56 ± 0.63 ^a^	7.88 ± 0.20 ^b^	9.63 ± 0.55 ^b^	29.51 ± 0.49 ^c^	26.03 ± 0.26 ^d^	27.97 ± 0.08 ^e^	20.42 ± 0.03 ^d^	23.37 ± 0.72 ^e^	18.50 ± 1.32 ^e^

^1^ Values are the mean ± standard deviation of 5 replicates. Means with different superscripts in each row differ significantly (*p* ≤ 0.05). ^2^ RMAS = raw makataan seed flour; RMOS = raw moringa seed flour; RESF = raw egusi seed flour; MOSH = moringa seed hydrocolloids; EGSH = egusi seed hydrocolloid; DMASF = defatted makataan seed flour; DMOSF = defatted moringa seed flour; MASH = makataan, hydrocolloid; DESF = defatted egusi seed flour.

**Table 2:** Proximate composition of makataan, egusi, and moringa seeds hydrocolloids.

	Hydrocolloid ^1^		
Proximate % *w*/*w* Dry Weight	Makataan	Egusi	Moringa
Protein	34.00 ± 2.65 ^a^	48.02 ± 1.00 ^b^	35.00 ± 1.00 ^a^
Moisture	7.80 ± 0.92 ^a^	7.19 ± 1.04 ^a^	7.71 ± 0.28 ^a^
Ash	4.09 ± 3.28 ^a^	6.57 ± 0.48 ^a^	5.98 ± 0.05 ^a^
Fat	4.53 ± 0.42 ^a^	0.53 ± 0.06 ^c^	5.88 ± 0.42 ^b^
Carbohydrate	29.51 ± 0.49 ^a^	23.37 ± 0.72 ^a^	29.57 ± 0.26 ^b^

^1^ Values are the mean ± standard deviation of 5 replicates. Means with a different superscript in each row differ significantly (*p* ≤ 0.05).

**Table 3:** Sugars in egusi, makataan, and moringa seed hydrocolloids ^1,2^.

		Hydrocolloids	
^1^ Sugar (g/g100)	^2^ MASH	MOSH	EGSH
Fructose	0.35 ± 0.02 ^a^	0.47 ± 0.01 ^b^	0.57 ± 0.78 ^c^
Glucose	0.49 ± 0.02 ^a^	3.05 ± 0.10 ^c^	0.78 ± 0.12 ^b^
Sucrose	4.95 ± 0.10 ^a^	7.68 ± 0.24 ^c^	5.90 ± 0.35 ^b^
Maltose	0.18 ± 0.00 ^a^	0.00 ± 0.00 ^a^	0.22 ± 0.02 ^c^
Raffinose	5.99 ± 0.01 ^a^	0.48 ± 0.02 ^a^	5.85 ± 0.21 ^b^
Stachyose	1.48 ± 0.02 ^a^	1.24 ± 0.02 ^b^	0.38 ± 0.03 ^a^

^1^ Values are the mean ± standard deviation. Means with a different superscript in each row differ significantly (*p* ≤ 0.05). ^2^ MOSH = moringa seed hydrocolloids; EGSH = egusi seed hydrocolloid; MASH = makataan seed hydrocolloid.

**Table 4:** Amino acids of makataan, moringa, and egusi seed hydrocolloids ^1,2^.

	Hydrocolloids ^2^		
^1^ Amino Acid	EGSH	MOSH	MASH
Non-essential g/100 g			
Arginine	5.99 ± 0.29 ^a^	4.69 ± 0.53 ^a^	7.25 ± 0.33 ^c^
Serine	1.08 ± 0.03 ^a^	0.78 ± 0.06 ^a^	1.59 ± 0.03 ^c^
Glycine	2.26 ± 0.09 ^a^	1.92 ± 0.12 ^a^	2.51 ± 0.17 ^b^
Asparagine	1.63 ± 0.13 ^a^	1.19 ± 0.03 ^a^	2.67 ± 0.26 ^c^
Glutamine	7.80 ± 0.39 ^a^	6.35 ± 0.08 ^a^	9.15 ± 0.61 ^c^
Alanine	1.17 ± 0.08 ^a^	0.99 ± 0.03 ^a^	1.58 ± 0.01 ^c^
Proline	1.15 ± 0.06 ^a^	1.48 ± 0.07 ^b^	1.40 ± 0.03 ^b^
Tyrosine	1.04 ± 0.09 ^a^	0.61 ± 0.12 ^a^	1.69 ± 0.25 ^c^
Essential			
Valine	0.79 ± 0.06 ^a^	0.75 ± 0.06 ^a^	1.30 ± 0.02 ^b^
Histidine	0.42 ± 0.02 ^a^	0.62 ± 0.14 ^b^	0.92 ± 0.01 ^c^
Leucine	1.39 ± 0.02 ^a^	1.25 ± 0.04 ^a^	1.13 ± 0.02 ^c^
Lysine	0.69 ± 0.17 ^a^	0.45 ± 0.14 ^a^	0.85 ± 0.38 ^a^
Methionine	1.13 ± 0.01 ^a^	0.75 ± 0.02 ^a^	1.58 ± 0.05 ^c^
Phenylalanine	1.07 ± 0.10 ^a^	0.92 ± 0.19 ^a^	2.09 ± 0.30 ^b^
Threonine	0.83 ± 0.05 ^a^	0.86 ± 0.04 ^a^	1.10 ± 0.02 ^b^

^1^ Values are the mean ± standard deviation. Means with a different superscript in each row differ significantly (*p* ≤ 0.05). ^2^ MOSH = moringa seed hydrocolloids; EGSH = egusi seed hydrocolloid; MASH = makataan seed hydrocolloid.

**Table 5:** Particle size and particle size distribution of moringa, makataan, and egusi hydrocolloids ^1,2^.

Hydrocolloid	D (0.5)	D [32]	Span	Uniformity
EGSH	437.00 ± 46.81 ^a^	461.48 ± 55.09 ^a^	0.64 ± 0.16 ^a^	0.18 ± 0.04 ^a^
MOSH	756.44 ± 33.45 ^b^	730.54 ± 31.99 ^b^	0.68 ± 0.01 ^a^	0.20 ± 0.01 ^b^
MASH	511.59 ± 0.46 ^a^	497.17 ± 35.45 ^a^	0.45 ± 0.01 ^a^	0.15 ± 0.00 ^a^

^1^ The values are the mean ± standard deviation. Different letter in the same column indicates significant differences (*p* < 0.05). ^2^ egusi seed hydrocolloid (EGSH), makataan seed hydrocolloid (MASH), and moringa seed hydrocolloid (MOSH).

**Table 6:** Change in particle size and size distribution of moringa, makataan, and egusi hydrocolloids after 10 s ^1,2^.

Hydrocolloids	^1^ D (0.5)	D [32]	D (0.5) 10 s	D [32] 10 s	Span
^2^ EGSH	437 ± 46.81 ^a^	461.48 ± 55.09 ^a^	12.31 ± 0.44 ^b^	10.60 ± 0.01 ^b^	2.33 ± 0.45 ^c^
MOSH	756.44 ± 33.45 ^a^	730.54 ± 31.99 ^a^	15.81 ± 0.45 ^b^	15.14 ± 0.05 ^b^	2.24 ± 0.55 ^c^
MASH	511.59 ± 0.46 ^a^	497.76 ± 0.50 ^a^	22.82 ± 1.45 ^b^	22.59 ± 0.45 ^b^	2,78 ± 0.56 ^c^

^1^ Values are the mean ± standard deviation of 5 replicates in different letters in the same column indicates significant differences (*p* < 0.05) ^2^ EGSH (egusi seed hydrocolloid), MASH (makataan seed hydrocolloid), and MOSH (moringa seed hydrocolloid).

**Table 7:** Energy dispersive X-ray of moringa, makataan, and egusi seed hydrocolloid ^1,2^.

	EGSH	MOSH	MASH
Carbon	59.3 ± 85.23 ^a^	57.29 ± 2.24 ^a^	51.55 ± 9.21 ^a^
Oxygen	34.01 ± 5.41 ^a^	37.20 ± 3.07 ^a^	35.90 ± 2.49 ^a^
Magnesium	0.58 ± 0.52 ^a^	0.540 ± 0.17 ^a^	0.43 ± 0.25 ^a^
Phosphorus	1.53 ± 0.35 ^a^	0.920 ± 0.31 ^a^	0.930 ± 84 ^a^
Sulphur	1.16 ± 0.59 ^a^	1.22 ± 1.04 ^a^	4.55 ± 0.98 ^b^
Potassium	1.11 ± 1.01 ^a^	2.68 ± 1.08 ^b^	4.28 ± 1.95 ^c^
Calcium	1.06 ± 1.43 ^a^	0.07 ± 1.12 ^a^	0.33 ± 0.52 ^a^

^1^ Mean ± standard deviation of 7 replicates. Different letters in the same row indicate significant differences (*p* < 0.05). ^2^ Egusi seed hydrocolloid (EGSH); makataan seed hydrocolloid (MASH); moringa seed hydrocolloid (MOSH).

**Table 8:** Colours in moringa, makataan, and egusi seed hydrocolloids ^1,2^.

Hydrocolloid	L*	a*	b*	C	H
Egusi	83.03 ± 0.23 ^a^	0.54 ± 0.08 ^b^	16.12 ± 0.14 ^c^	16.13 ± 0.16 ^c^	87.99 ± 0.20 ^a^
Moringa	81.39 ± 0.04 ^a^	0.61 ± 0.01 ^b^	17.65 ± 0.00 ^c^	17.66 ± 0.00 ^c^	87.03 ± 0.02 ^a^
Makataan	78.63 ± 0.10 ^a^	0.90 ± 0.02 ^b^	16.56 ± 0.13 ^c^	16.50 ± 0.09 ^c^	86.87 ± 0.06 ^d^

^1^ Mean ± standard deviation in five replicates. Means with different superscripts in the same column indicate significant differences (*p* < 0.05). ^2^ EGSH (egusi seed hydrocolloid); MASH (makataan seed hydrocolloid): MOSH (moringa seed hydrocolloid).

**Table 9:** Equilibrium interfacial tensions ^1,2^.

Emulsifier Type	Emulsifier (%) ^2^	γ , (mN/m)
Lecithin	10	9.30 ^a^
^2^ EGSH	10	3.67 ^b^
MASH	10	4.84 ^b^
MOSH	10	12.16 ^c^
Guar	10	4.04 ^b^

^1^ Mean ± standard deviation of different superscripts in the same column indicate significant (*p* < 0.05) differences. ^2^ EGSH (egusi seed hydrocolloid); MASH (makataan seed hydrocolloid): MOSH (moringa seed hydrocolloid).

## Data Availability

The original contributions presented in the study are included in the article, further inquiries can be directed to the corresponding author.
